# Effect and potential risks of using multilevel cement-augmented pedicle screw fixation in osteoporotic spine with lumbar degenerative disease

**DOI:** 10.1186/s12891-020-03309-y

**Published:** 2020-04-28

**Authors:** Yong-chao Tang, Hui-zhi Guo, Dan-qing Guo, Pei-jie Luo, Yong-xian Li, Guo-ye Mo, Yan-huai Ma, Jian-cheng Peng, De Liang, Shun-cong Zhang

**Affiliations:** 1 Spine Surgery Department, The First Affiliated Hospital of Guangzhou University of Chinese Medicine, Guangzhou, 510407 China; 2 The 1st Institute of Clinical Medicine, Guangzhou University of Chinese Medicine, 12 Airport Road, Baiyun District, Guangzhou, 510405 Guangdong People’s Republic of China

**Keywords:** Multilevel fixation, Cement-augmented pedicle screw instrumentation, Lumbar degenerative disease

## Abstract

**Background:**

The increase of augmented level and bone cement dose are accompanied by the rising incidence of cement leakage (CL) of cement-augmented pedicle screw instrumentation (CAPSI). But the effect and potential risks of the application of CAPSI to osteoporotic lumbar degenerative disease (LDD) have not been studied in the case of multilevel fixation. This study aimed to investigate the effectiveness and potential complications of using multilevel CAPSI for patients with osteoporotic LDD.

**Methods:**

A total of 93 patients with multilevel LDD were divided into the CAPSI group (46 subjects) and the conventional pedicle screw (CPS) group (47 subjects), including 75 cases for three levels and 18 cases for four levels. Relevant data were compared between two groups, including baseline data, clinical results, and complications.

**Results:**

In the CAPSI group, a total of 336 augmented screws was placed bilaterally. The CL was observed in 116 screws (34.52%). Three cemented screws (0.89%) were found loosened during the follow-up and the overall fusion rate was 93.47%. For perioperative complications, two patients (4.35%) experienced pulmonary cement embolism (PCE), one patient augmented vertebral fracture, and three patients (6.52%) wound infection. And in the CPS group, thirty-three screws (8.46%) suffered loosening in cranial and caudal vertebra with a fusion rate of 91.49%. The operation time and hospital stay of CAPSI group were longer than the CPS group, but CAPSI group has a lower screw loosening percentage (*P*<0. 05). And in terms of blood loss, perioperative complications, fusion rate, and VAS and ODI scores at the follow-up times, there were no significant differences between the two groups.

**Conclusions:**

Patients with osteoporotic LDD underwent multilevel CPS fixation have a higher rate of screw loosening in the cranial and caudal vertebra. The application of cemented pedicle screws for multilevel LDD can achieve better stability and less screw loosening, but it also accompanied by longer operating time, higher incidence of CL, PCE and wound infections. Selective cement augmentation of cranial and caudal pedicle screws may be a worthy strategy to decrease the complications.

## Background

Pedicle screw fixation is widely used in degenerative lumbosacral disease, secondary kyphosis or deformity, and thoracolumbar fractures due to a variety of indications, such as bony fusion promotion, deformity correction, and fixation for vertebral fractures [1–3]. Although it is the gold standard for the treatment of degenerative and traumatic spinal diseases [2], the purchase strength of it in low-quality bone still reduced significantly [4, 5]. Instrument failure, such as screw loosening and back-out, associated with nonunion, pseudoarthrosis, and progressive kyphosis, is the most common complication of posterior internal fixation in osteoporosis patients [6, 7].

BMD plays a pivotal role in the stability of the pedicle screw [8], which can be also affected by other factors include age, smoking, diabetes, different spinal diseases, fusion methods, fixation segments, sacral fixation, repeated screw placement, spinopelvic parameters, and geometry shape of the screw [9–12]. Studies have shown that the rate of screw loosening is 1–15% in non-osteoporotic patients, but up to 10–60% in osteoporotic patients [9, 10] and even 50–62.8% in multilevel fixation [11–13]. Since both osteoporosis and multilevel lumbar degenerative disease (LDD) are common disorders in elderly patients [2], improving the attachment of internal instruments for patients with multilevel osteoporotic LDD has become an essential mission for spine surgeons.

Both fenestrated and solid screws with cement augmentation have been proven effective in improving the pullout strength of screws and reducing the risk of fixation failure in biomechanical studies and clinical trials [14, 15]. However, CL remains a common complication of CAPSI, and the leakage rate is 11.6–82.4% [16, 17]. CL may cause severe complications such as nerve injury, vascular damage, pulmonary cement embolism (PCE), cardiac embolism, and anaphylactic shock [17, 18]. Multilevel cement-augmented pedicle screws in osteoporotic bone are considered to lead to a higher incidence of CL and cement-related complications. However, no study has selectively analyzed the effect and potential risks of multilevel CAPSI in osteoporotic spine with LDD. Therefore, this study aimed to compare the clinical and radiological results of osteoporotic patients with multilevel LDD (three or four levels) who were treated with CAPSI with those who were treated with CPS.

## Methods

### Patient population

From February 2010 to February 2017, all patients visiting our hospital for CAPSI or CPS were recruited. The inclusion criteria were as follows: (1) patients diagnosed with LDD by symptoms, signs, and imaging examinations who were not responsive to at least 3 months of conservative treatment, (2) those with posterior interbody fusion using consecutive three-level (L2–L5 or L3–S1) or four-level (L1–L5 or L2–S1) pedicle screw implantation, and (3) those who had undergone lumbar vertebral bone mineral density (BMD) measurement using dual-energy X-ray absorptiometry and with T ≤ − 2.5 SD. The exclusion criteria were as follows: (1) patients with vertebral fracture, tumor, or infection, (2) those with a history of lumbar surgery, (3) those with partial lumbar screws that are augmented in the CAPSI group, or (4) those who had incomplete imaging data at follow-up.

**Fig. 1:**
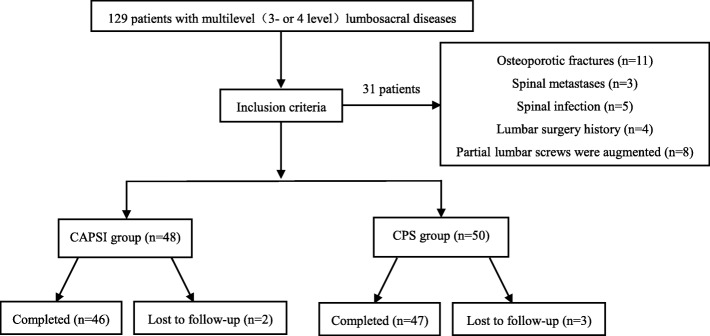
Diagram showing the process of patient selection

Ninety-three patients, including 75 three level cases (L2–L5: 21 cases, L3–S1: 54 cases) and 18 four level cases (L1–L5 2 cases, L2–S1 16 cases) with an average follow-up of 35.61 ± 19.56 (range: 24–108) months, were divided into the CAPSI group (46 patients) and the CPS group (47 patients). In the CAPSI group, all patients had received cement-augmented screws at the lumbar vertebra, but only 8 patients received augmented screws and 27 received conventional pedicle screws at S1 because of the lack of technique for sacral pedicle screw with cement augmentation, which would be used later. The CAPSI group had 7 men and 39 women with a mean age of 70.65 ± 7.20 (range: 52–86) years, T scores of − 3.18 ± 0.94 (range: − 2.5 to − 4.9) SD, and follow-up of 31.87 ± 15.49 (range: 24–108) months. Twenty-five patients had lumbar spinal stenosis with spinal instability, 9 had lumbar spondylolisthesis, and 12 had lumbar spinal stenosis with degenerative scoliosis. Solid screws were used in 16 patients and fenestrated screws were used in 30 patients. The CAP group had 6 males and 41 females with a mean age of 67.91 ± 7.62 (range: 55–86) years, T scores of − 2.89 ± 0.49 (range: − 2.5 to − 5.0) SD, and follow-up of 35.53 ± 21.54(range: 24–96) months. Thirty patients had lumbar spinal stenosis with spinal instability, 8 had lumbar spondylolisthesis, and 9 had lumbar spinal stenosis with degenerative scoliosis. A flowchart of this study is shown in Fig. 1.

### Surgical technique

All patients underwent standard posterior lumbar interbody fusion (PLIF) and/or transforaminal lumbar interbody fusion (TLIF) under general anesthesia. The decision for cement augmentation was made by an experienced surgeon after evaluating the patients’ BMD and the biomechanical stability of the implanted pedicle screw. The operation for installation of a traditional pedicle screw was performed using a standard technique according to the literature [4, 10]. The procedure for placement of fenestrated pedicle screw with cement and solid pedicle screw with pre-augmented trajectory has been described in our previous study [19]. In general, pedicle screws with a diameter and length of 6.5 mm and 45 mm, respectively, were used in the lumbar spine, and those with a diameter and length of 6.5 mm and 40 mm, respectively, were used in the sacrum. For every lumbar pedicle screw and sacral pedicle screw, approximately 1.5–4 mL and 1.5–2.5 mL of polymethylmethacrylate (PMMA) was injected, respectively.

At the early stage after surgery, the patients were required to perform lower limb exercises on the bed. The drainage tube was removed when the amount of drainage was < 50 mL/d. All patients began to walk with protection on the waist 3 or 4 days after the operation. Patients were required to wear a waist protector for the first month after surgery. Routine postoperative radiography of the thorax was performed in augmented patients, and patients complaining of discomfort in the heart or lung received additional thoracic CT. All patients received anti-osteoporosis treatment—calcium carbonate, vitamin D3, and bisphosphonate—throughout the treatment period.

### Evaluation indicators

The visual analog scale (VAS) and the Oswestry Disability Index (ODI) at different follow-up periods were recorded to assess clinical outcomes. Procedure duration, intraoperative blood loss, and duration of hospitalization of the two groups were abstracted from medical records. Major postoperative complications were also compared between the two groups, including nerve injury, dural tearing, wound infection, and revision surgery.

Radiologic evaluation included spinopelvic parameters, screw loosening rate, and lumbar fusion status. Two experienced orthopedists were assigned to evaluate the radiologic parameters independently. All data were measured twice by two researchers, and the mean values were used for analysis. The degree of lumbar lordosis (LL), pelvic tilt (PT), and pelvic incidence (PI) were assessed as previously described [20]. Screw loosening was defined as a halo sign showing a radiolucent line of ≥1 mm around the screw in X-ray or CT images on one or both sides after the surgery [11, 21]. Solid fusion was determined according to the criterion described by Dakhil et al. [22] as a bridging interbody bone occurring in the operated segment and an angle of < 5° on dynamic X-ray images at the fixed level.

### Statistical method

The data were analyzed using the SPSS 19.0 software (IBM, Inc., Armonk, NY, USA). The difference of the continuous variables between the two groups was compared by using the independent sample *t*-test (age, BMD, BMI, VAS and ODI scores, follow-up, operation time, blood loss, and hospital stay and spinopelvic parameters). The chi-square test was used to compare the classification variables (gender, diabetes, hypertension, smoking, diagnosis, fusion methods, bone graft, fusion level, lumbosacral fixation, different patterns of S1 pedicle screw placement, lumbosacral fixation, complications, screw loosening, nonunion patients). A *P* < 0.05 was considered statistically significant.

## Results

### Baseline characteristics

There were no statistical differences between these two groups in terms of sex, age, BMD, BMI, diabetes, hypertension, smoking, diagnosis, fusion methods, bone graft, fusion level, lumbosacral fixation, different patterns of S1 pedicle screw placement, lumbosacral fixation, preoperative VAS score, preoperative ODI score, follow-up, preoperative LL, preoperative PT, and preoperative PI (Table 1).

**Table 1:** Comparison of baseline data in two groups

Factors	CAPSI group (*n* = 46)	CPS group (*n* = 47)		*P* value
Male/female(n)	7/39	6/41	x^2^ = 0.116	0.733
Age (year)	70.65 ± 7.20	67.91 ± 7.62	t = 1.780	0.078
Body mass index	23.91 ± 3.36	23.64 ± 2.85	t = 0.411	0.682
Bone mineral density	−3.18 ± 0.94	−2.89 ± 0.49	t = −1.822	0.072
Diabetes(n)	12	9	x^2^ = 1.033	0.333
Hypertension(n)	23	22	x^2^ = 0.095	0.837
Smoking(n)	3	2	x^2^ = 0.284	0.671
Diagnosis (n)			x^2^ = 0.933	0.653
Lumbar spinal stenosis	25	30		
Spondylolisthesis	9	8		
Degenerative scoliosis	12	9		
Fusion methods(n)			x^2^ = 0.732	0.665
TLIF	26	28		
PLIF	5	7		
PLIF+TLIF	15	12		
Bone graft			x^2^ = 0.665	0.496
Autograft bone	15	12		
Autograft and allograft bones	31	35		
Fusion level(n)			x^2^ = 3.517	0.522
L2-L5	9	12		
L3-S1	26	28		
L1-L5	2	0		
L2-S1	9	7		
Lumbosacral fixation(n)	35	35	x^2^ = 0.033	1.000
S1 bicortical/tricortical fixation(n)	14/21	19/16	x^2^ = 1.438	0.338
VAS score	7.19 ± 0.86	7.34 ± 0.73	t = −0.876	0.383
ODI score(%)	52.04 ± 7.58	51.11 ± 7.46	t = 0.601	0.549
Follow-up time(months)	31.87 ± 15.49	35.53 ± 21.54	t = −0.94	0.350
Pre-operation LL(°)	31.19 ± 18.27	35.68 ± 13.53	t = −1.350	0.180
Pre-operation PT(°)	23.50 ± 10.49	19.76 ± 8.96	t = 1.850	0.068
Pre-operation PI(°)	48.07 ± 12.61	51.30 ± 13.54	t = −1.189	0.237

CAPSI Cement-augmented pedicle screw instrumentation, CPS conventional pedicle screw, TLIF transforaminal lumbar interbody fusion, PLIF posterior lumbar interbody fusion, VAS Visual Analogue Scale, ODI Oswestry Disability Index, LL lumbar lordosis, PT Pelvic title, PI Pelvic incidence

### Clinical effects and complications

**Fig. 2:**
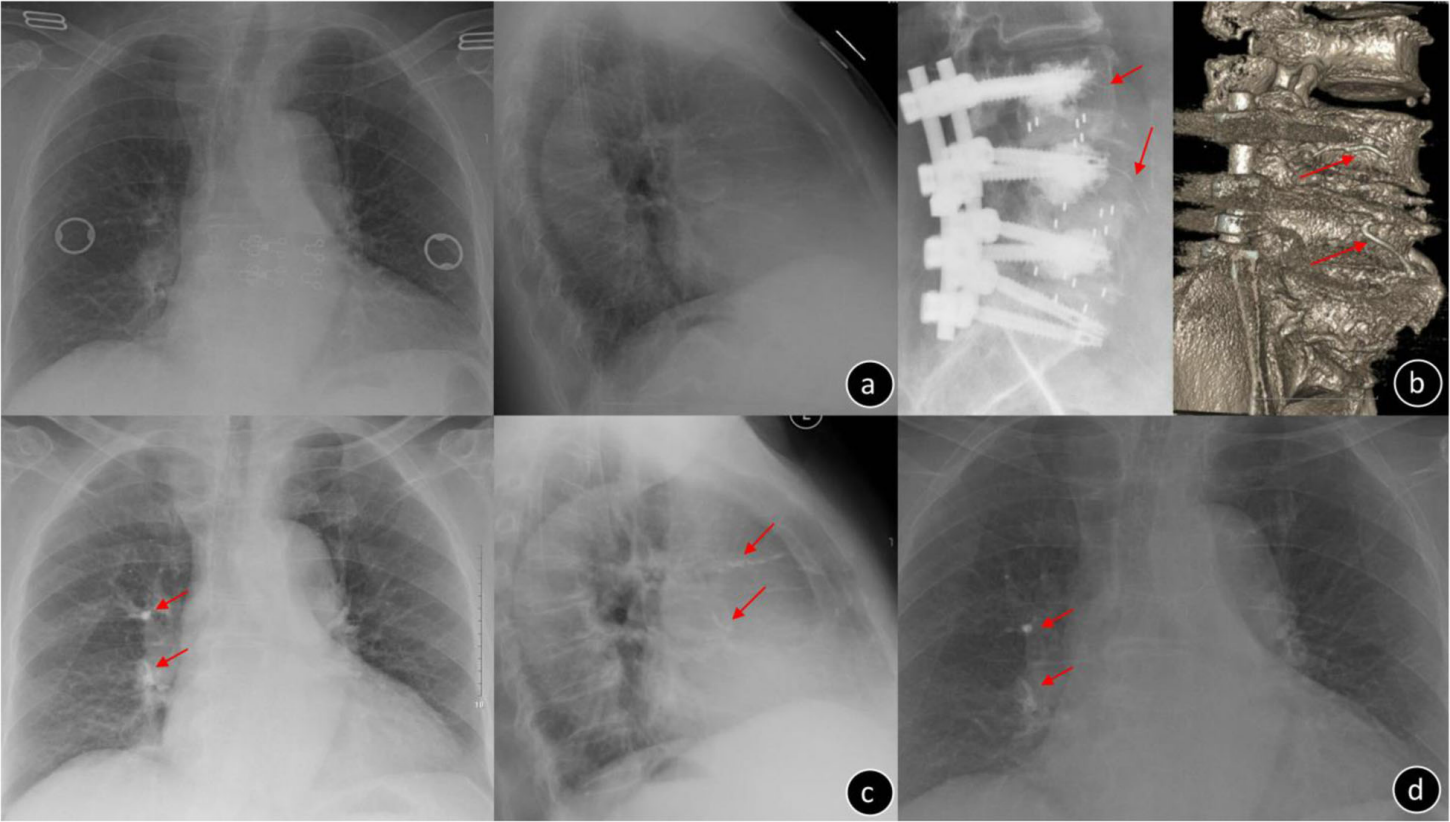
Female, 73-year-old, T = −3.5 SD **a.** Preoperative chest x-ray image **b**. Postoperative x-ray and CT scans illustrating CL to the segmental vein in L3/L4 (arrow) **c**. The postoperative anteroposterior radiograph showed pulmonary cement embolism on the right side (arrow); this patient experienced post-op dyspnea and blood oxygen desaturation and recovered within 6 days after oxygen inhalation and anticoagulation treatment **d**. Two years after the operation, the chest x-ray showed cement embolism was in situ (arrow) and the patient was asymptomatic

**Fig. 3:**
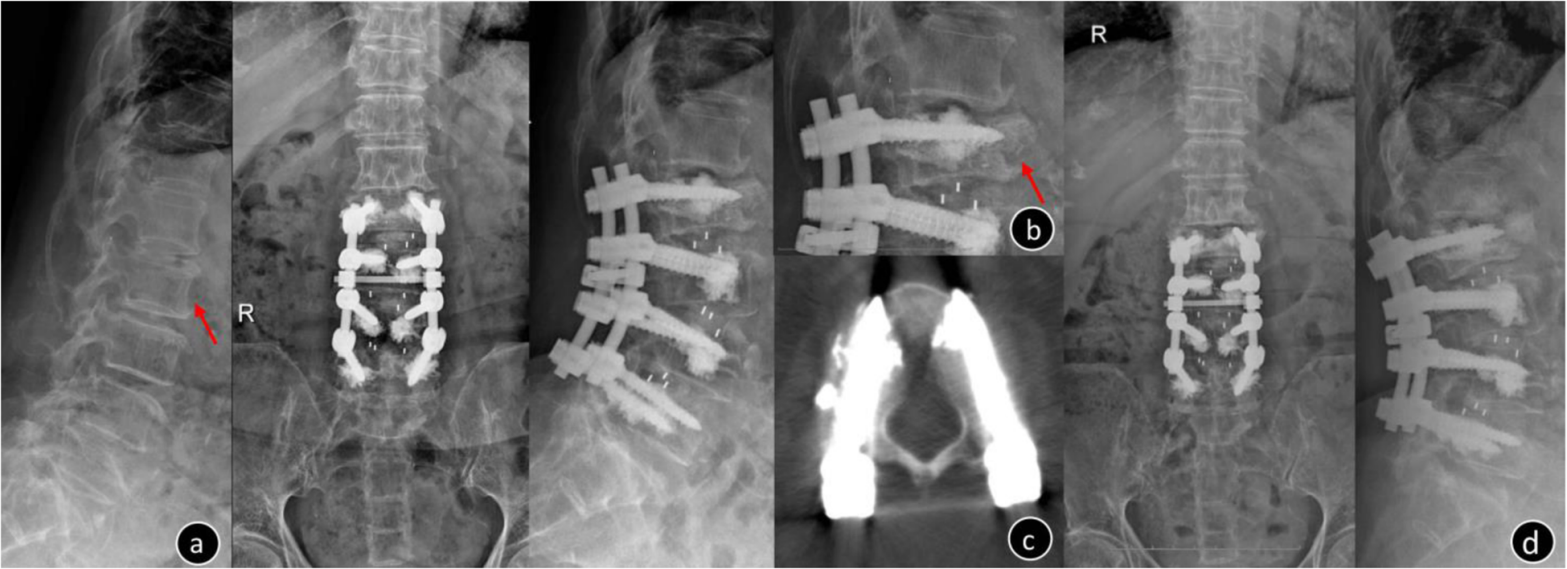
Female, 73-year-old, T = − 3.5 SD, the patient underwent solid pedicle screw with a pre-augmented trajectory **a**. The preoperative x-ray image showed lumbar vertebral degeneration and no fracture was found in the L2 vertebral; **b**. Postoperative x-ray: L2 vertebral fracture; **c**: when a screw was implanted late and the cement becomes solidified, the screw may deviate augmented trajectory, and the following rupture of the cortex bone may result in vertebral fracture; **d**. Three years after the operation, the x-ray showed that L2 vertebral body collapsed, the fracture healed and the L2 pedicle screw has not loosened

**Fig. 4:**
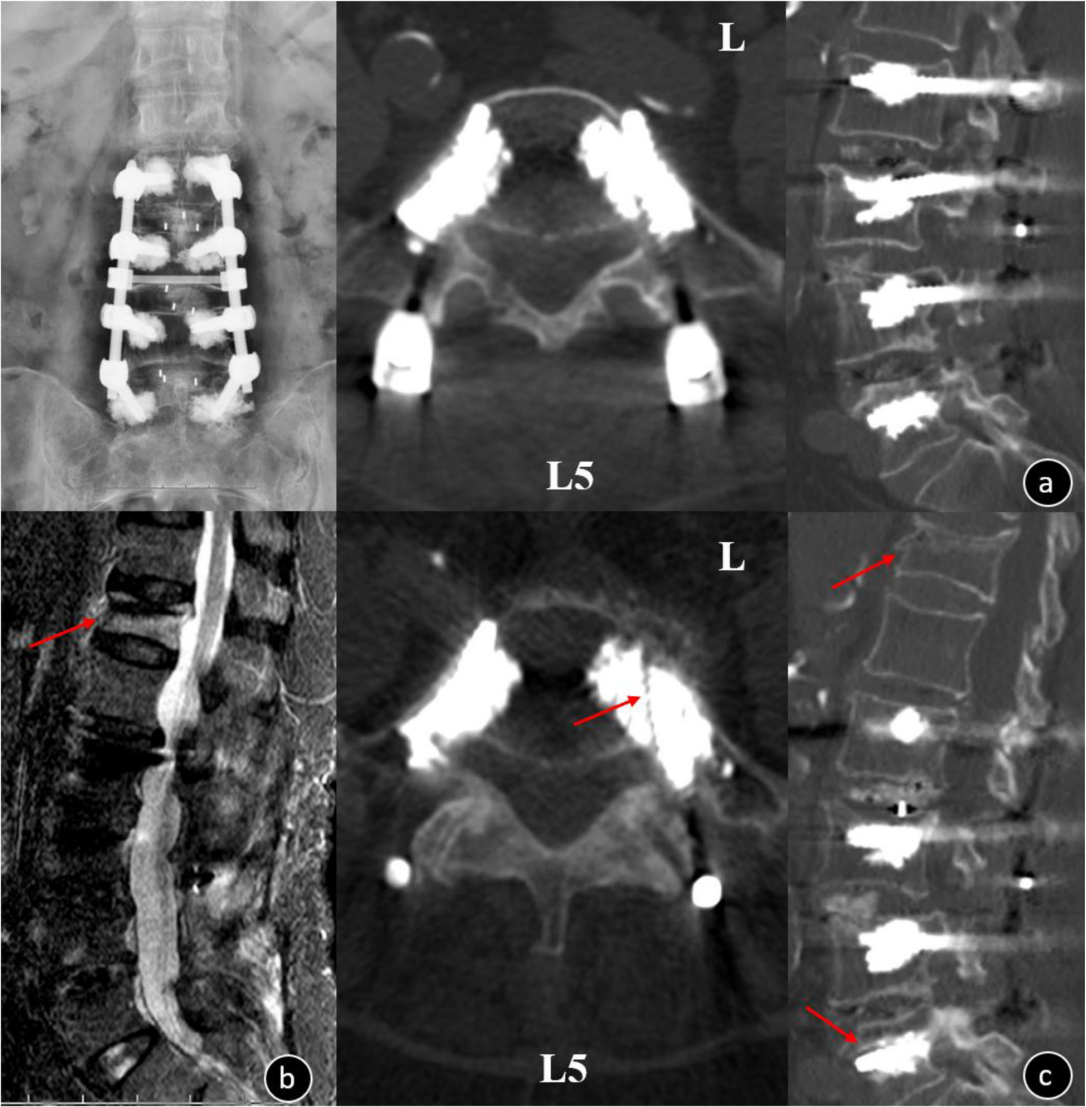
Female, 69-year-old, T = − 3.4 SD **a**. the post-operation X-rays and CT showed the internal fixation was in good position and there was no screw loosening in L5 **b**. Seven months after the operation, the patient accidentally fell, MRI showed T12 acute osteoporotic vertebral compression fracture; **c**. the CT illustrated the pedicle screw was loosened in left L5 (arrow). The reason of screw loosening may be related to long segment fixation and stress concentration on the tail of the fixed segment when tumbled

**Fig. 5:**
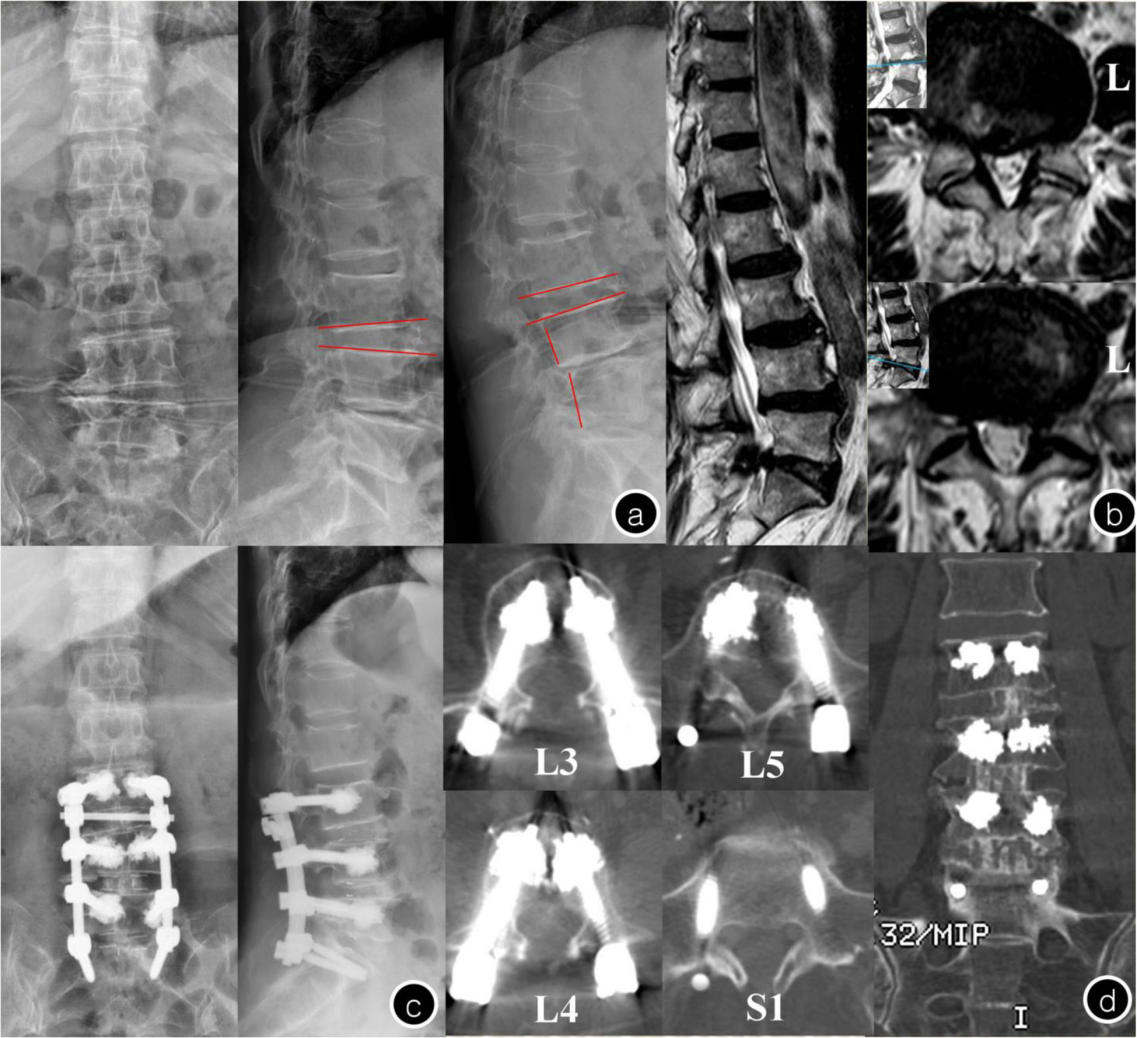
Female, 75-year-old, T = -3.8 SD **a**. Preoperative x-ray image showed lumbar vertebral degeneration with spinal instability in L3-L5; **b**. Preoperative MRI: L4/5 spinal canal stenosis and L5-S1 level lumbar disk herniation; **c**. Postoperative x-ray: the internal fixation was in good position and without serious leakage of cement; **d**. Five years after the operation, the CT showed that pedicle screw has not loosened and the L3-S1 was interbody fusion

There were 336 cement-augmented pedicle trajectories used, with a mean of 7.30 ± 1.4 (range: 6–10) for the instrumented screw and 1.85 ± 0.65 mL (range: 1–4) for PMMA per pedicle screw. CL was observed in 44 patients (95.65%) and 116 screws (34.52%), and CL most often occurred in the perivertebral venous system; leakage via segmental veins and basivertebral veins was seen in 109 screws (93.97%). Perioperative complications included the following: PCE (2 patients, of whom 1 was symptomatic and the other was asymptomatic; Fig. 2), hypotension (2 patients), superficial skin infection that required surgical debridement (2 patients), deep infection but the instrumentation was not removed (1 patient), dural tear and discharged after surgical suture repair (1 patient), augmented vertebral fracture (1 patient; Fig. 3), radiating leg pain (6 patients), and discomfort at the last follow-up (2 patients). There were no serious complications caused by the leakage of bone cement, such as nerve damage, cardiac embolism, or death. Three cemented screws (0.89%) (Fig. 4) and 24 S1 conventional screws (44.44%) were found to be loose at the follow-up. Of the 3 patients who had nonunion, 2 required revision surgery with a fusion rate of 93.47%. During follow-up, adjacent fractures were observed in 6 patients and vertebral fractures were observed in 8 patients. Symptomatic adjacent disc degeneration was observed in 3 patients, of which 2 required revision surgery. A typical case of the CAPSI group is shown in Fig. 5.

**Fig. 6:**
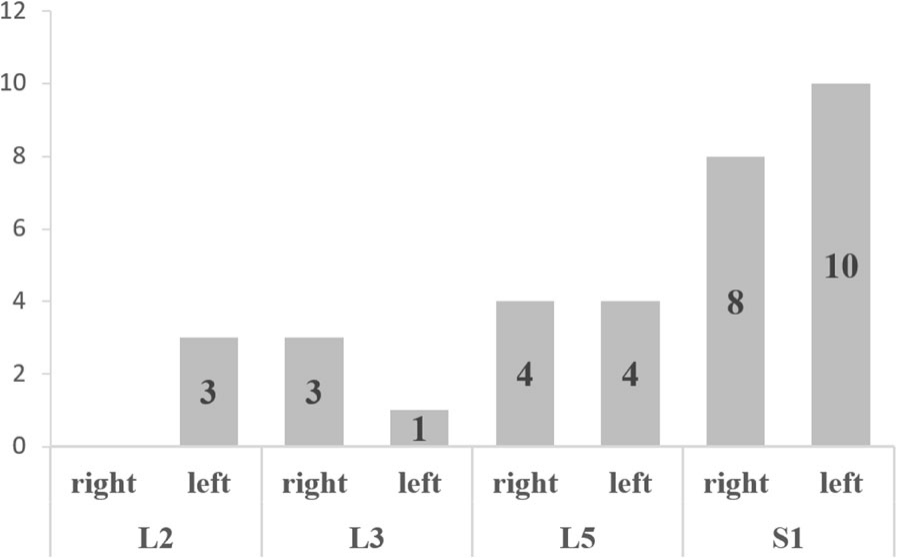
The distribution of screws loosening in the CPS group

**Fig. 7:**
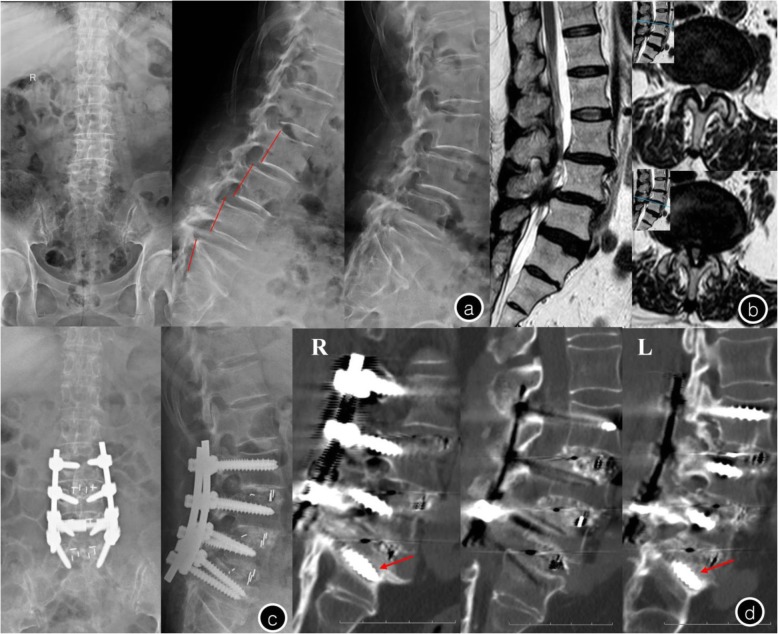
Female, 73-year-old, T = −2.5 SD **a**. Preoperative X-rays showed scoliosis in lumbar spine and L3-L5 spondylolisthesis; **b**. Preoperative MRI showed L3–5 level lumbar disk herniation with spinal canal stenosis; **c**. The lumbar anterior-posterior radiographs after surgery showed good reduction of spondylolisthesis; **d**. One year after the operation, CT illustrated halo sign appeared around S1 screws (arrows) and the S1 pedicle screw has loosened in bilateral. Meanwhile, L3-S1 was interbody fusion

In the CPS group, perioperative complications included the following: hypotension (1 patient), dural tear (1 patient), and severe radiating leg pain due to pedicle screw misplacement (8 patients, of which 3 still complained about discomfort at the last follow-up). Seventeen patients (36.17%) and 33 screws (8.46%) showed loosening in the cranial or caudal vertebra (Fig. 6). Of the 4 patients who experienced nonunion, 1 required revision surgery with a fusion rate of 91.49%. Adjacent fractures were observed in 3 patients and 3 vertebrae during follow-up, and symptomatic adjacent disc degeneration was observed in 3 patients—all required revision surgery. A typical case of the CPS group is shown in Fig. 7.

**Fig. 8:**
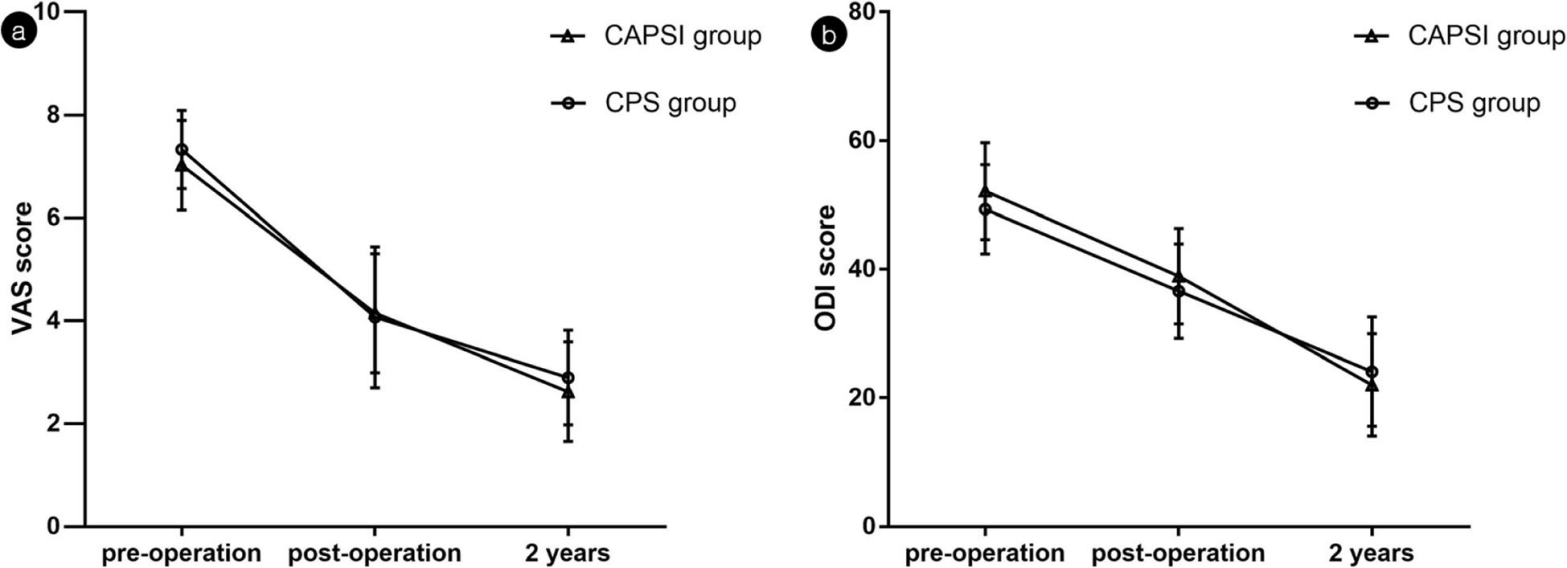
Changes in clinical results between the two groups during follow-up period, **a**. Mean Visual Analog Scale (VAS) score; **b**. Mean Oswestry Disability Index (ODI) score

Compared with the date of preoperation, a larger postoperation LL was found in both the CAPSI and CPS groups. However, there were no statistical differences in preoperative and postoperative PT and PI both groups. Longer operation time, longer hospital stay, and less screw loosening were found in the CAPSI group than in the CPS group (*P* < 0.05). In terms of blood loss, perioperative complications, fusion rate, VAS and ODI scores at follow-up, postoperative LL, postoperative PT, and postoperative PI, no significant difference was detected between the two groups (Table 2 and Fig. 8).

**Table 2:** Comparison of the operation and clinical effects of two groups

Factors	CAPSI group(*n* = 46)	CPS group(*n* = 47)		*P* value
Operation time(min)	303.07 ± 61.25	268.72 ± 64.46	t = 2.633	0.010
Blood loss(ml)	1176.09 ± 763.82	1072.34 ± 838.46	t = 0.485	0.629
Hospital stay(days)	24.54 ± 8.82	20.85 ± 8.78	t = 2.023	0.046
Complications (n)	15	10	x^2^ = 1.526	0.249
Post-operation LL(°)	39.59 ± 10.55^a^	41.57 ± 9.27^a^	t = −0.958	0.341
Post-operation PT(°)	21..47 ± 10.18	17.92 ± 10.14	t = 1.684	0.096
Post-operation PI(°)	49.47 ± 12.59	52.03 ± 12.81	t = −0.984	0.327
Screw loosening(n)	3/336	33/390	x^2^ = 23.996	0.000
Nonunion patients(n)	3	4	x^2^ = 0.133	1.000
Post-operation VAS	4.19 ± 1.13^a^	4.02 ± 1.39^a^	t = 1.973	0.052
Post-operation ODI	39.30 ± 7.11^a^	36.29 ± 7.58^a^	t = 0.663	0.509

^a^ Significantly greater than the value found in preoperation

*CAPSI* Cement-augmented pedicle screw instrumentation, *CPS* Conventional pedicle screw, *LL* Lumbar lordosis, *PT* Pelvic title, *PI* Pelvic incidence, *VAS* Visual Analogue Scale, *ODI* Oswestry Disability Index

## Discussion

Pedicle screw fixation in osteoporotic spine is gradually becoming common because of the aging population. Therefore, we will have to face the increasing incidences of pedicle screw loosening and instrumentation-associated complications [23]. According to the literature, 17% of revision surgeries are associated with pedicle screw failure [5]. Thus, CAPSI has been widely used for patients with poor bone quality due to its reliable biomechanical stability [15, 24–26]. But the treatment of multilevel LDD with cement-augmented pedicle screws is more complicated and challenging compared with 1 or 2-level LDD because of the increase of CL and mechanical overload [27]. Many studies either assessed only the results of short segment augmentation or assessed the results of long and short segment augmentation together, and most data are not related to osteoporotic LDD. So far there is currently little data about the effectiveness and potential risks of CAPSI treating the multilevel LDD.

Although the application of augmented pedicle screws for multilevel LDD can achieve better stability and less screw loosening, it also carries a high risk of CL, PCE, and wound infections. The CL rate of the present study was 34.52%. Several studies indicated that CAPSI has a high risk of asymptomatic cement leakage and serious complications were also reported in the literature. Methods to reduce CL in LDD include: (1) Using high viscosity cement or toothpaste viscosity cement [28]. (2) Reducing the volume of cement and the number of augmented screws [29, 30]. Experimental studies have indicated that screw stability does not significantly improve when the volume of PMMA exceeded 2.8 ml each screw [25]. Therefore, the volumes of bone cement should be optimized according to the severity of osteoporosis. (3) Planning the trajectory and the size of pedicle screws by preoperative CT to achieve the accurate placement of pedicle screws. Our previous study has found that a smaller distance between the tip of screw and the midline of vertebra is closely related to the epidural CL [19]. (4) We suggest injecting cement with small doses and slow speed (the cement was injected by every 0.1 ml increment in this study) or creating a small cavity in the vertebral body prior to the cement injection [29]. (5) The injection of cement should be confirmed consecutively by fluoroscopy.

In the present study, PCE was detected in two cases (4.35%) that one patient was symptomatic. Clinical research has shown the incidence rate of pulmonary embolism ranged from 3.5 to 23% after percutaneous vertebroplasty and kyphoplasty [17, 31]. Most of the pulmonary embolism cases are asymptomatic while the incidence of symptomatic is about 1.4% ~ 4.1% [31]. Severe PCE is rare but fatal [17, 32]. The optimal treatment strategy for PCE remains controversial. Several researches have suggested that for asymptomatic PCE, it is feasible to adopt clinical observation and regular follow-up without anticoagulants. And for symptomatic PCE, long-term anticoagulation therapies, including initial heparin during hospitalization and oral coumarin therapy, can prevent cardiovascular complications. Surgery for removing cement embolus is also an alternative strategy for patients with severe embolism [17].

Wound infection may lead to nonunion and instrumentation removal. The infection rate of CAPSI group was very high (6.52%, 3/46). We believe it was related to advanced age, long operation time, large intraoperative bleeding, and other comorbidities, such as diabetes. The average age of these patients was 78 years old, average operation time 326.67 min, average blood loss 1433.33 ml, and two of them have diabetes. Few clinical studies reported augmented screw loosening, but it can’t mask the fact that it is one of the non-ignorable complications in CAPSI. Two patients (three augmented screws) were found loosening in our study, one of the screws is caused by tumble and the others poor distribution of bone cement around them. Due to the worse balance control, lower extremity pain, and musculoskeletal degeneration, LDD patients become more prone to fall, even after spinal surgery [33]. When bone cement was poorly-dispersed around the pedicle screw, the biomechanical stability were dramatically lower than those well distributed one [14, 23]. In addition, according to our experience, when a solid screw with PMMA augmentation is implanted late and cement became solid, the sclerotic wall of the cement does not allow this screw to interdigitate into the PMMA, and thus the screw may deviate from augmented trajectory.

We attempted to inject PMMA and then placed screws immediately for multilevel augmentation from the beginning, but it brought a risk that screws cannot interdigitate into sclerotic cement (Fig.3). Later we found using fenestrated screws leads to better control of PMMA injection (especially when more than two vertebra need to be augmented). Thus, we propose using fenestrated screws for multilevel augmentation, especially for inexperienced surgeons. But we tend to insert solid screws in S1 to reduce the leakage caused by fenestrated screws penetrating the presacral bone cortex [20]. Otherwise, we usually use augmented screws on two vertebral bodies at a time rather than three bodies, which minimizes the difficulty of operation and facilitates C-arm fluoroscopy.

Screw loosening of multilevel fixation often occurs in the cranial and caudal segment and far more frequently in the caudal (S1 in particular). Consistent with previous studies [9, 27], twenty-four S1 conventional screws (44.44%) were found loosening in the CAPSI group, and thirty-three screws (8.46%) in CPS group, including seven screws at cranial level and twenty-six at caudal level (eight in L5, eighteen in S1). Wu et al. [9] analyzed 658 screws of 126 patients and found that 25 patients experienced screw loosening, 18 patients (72%) had loosened screws in caudal vertebra while 7 (28%) in the cranial vertebra. Li et al. [27] also found that the failure of internal fixation usually develops at the caudal level in cases of multilevel pedicle screw fixation. Therefore, we recommend increasing properly the bone cement dose in cranial and caudal screw to reduce the need for sacroiliac screw fixation and other pelvic fixation [20].s.

Since the application of pedicle screws with cement on all segments will increase the operating time, CL, PCE, and incision infection, only cementing the selected strategic vertebrae, such as cranial or caudal pedicle screw alone, could be a worthy strategy. Based on the clinical and radiological data of 31 patients treated with cement injections only for the selected strategic vertebrae and augmentation for all segments, Erdem [34] found the rate of PCE and operative time were markedly higher in all segments cement injections group, but there is no significant difference in screw loosening and clinical efficacy between two groups. Therefore, selective cement augmentation for both cranial and caudal pedicle screws could be a potential optimal procedure to decrease the side complications of CAPSI.

There are several limitations of our study. First, since we have not analyzed other risk factors of the screw loosening and fusion, such as the strength of paravertebral muscles, single or double cage, the length/diameter of screw and the repeated placement of screws during operation, as well as the specific time of screw loosening and asymptomatic adjacent segment degeneration. Second, as a retrospective study, it is difficult for us to control the uniformity of the patients and there is a degree of selective bias in this study, such as the inclusion of cases, the grouping of patients, and the selection of surgical methods. In addition, the actual rate of loosening may be lower than those reported in this paper, because the patients with screw loosening are more likely to receive follow-up, while patients with good curative effect may be reluctant to return to the hospital.

## Conclusions

Patients with osteoporotic LDD underwent multilevel CPS fixation have a high rate of screw loosening in the cranial and caudal level. The application of augmented pedicle screws on multilevel LDD can achieve better stability and less screw loosening, but it also accompanied by longer operating time, higher incidence of CL, PCE and wound infections. Selective cement-augmenting cranial and caudal pedicle screws may be a worthy strategy to decrease the complications.

## Data Availability

The datasets used in the current study are not publicly available due to privacy protection but are available from the corresponding author on reasonable request.

## Abbreviations

CAPSI: Cement-augmented pedicle screw instrumentation
CL: Cement leakage
LDD: Lumbar degenerative disease
CPS: Conventional pedicle screw
PCE: Pulmonary cement embolism
BMD: bone mineral density
BMI: Body mass index
VAS: Visual analogue scale
ODI: Oswestry disability index
PMMA: Polymethylmethacrylate
CT: Computed tomography
TLIF: Transforaminal lumbar interbody fusion
PEEK: Polyether ether ketone
LL: Lumbar lordosis
PT: Pelvic title
PI: Pelvic incidence
